# A Review on Current Notion in Frozen Shoulder: A Mystery Shoulder

**DOI:** 10.7759/cureus.29362

**Published:** 2022-09-20

**Authors:** Pratik Phansopkar, Moh'd Irshad Qureshi

**Affiliations:** 1 Musculoskeletal Physiotherapy, Ravi Nair Physiotherapy College, Datta Meghe Institute of Medical Sciences, Wardha, IND; 2 Neuro-Physiotherapy, Ravi Nair Physiotherapy College, Datta Meghe Institute of Medical Sciences, Wardha, IND

**Keywords:** review, mirror therapy, joint mobilization, medical therapy, physical therapy, frozen shoulder

## Abstract

Frozen shoulder (FS) is a common condition affecting the population between the ages of 30 and 60; the causative agent is idiopathic, sedentary lifestyle, post-traumatic, or secondary to any pathological conditions. The pathology of FS is characterized by cytokine-mediated synovial inflammation with fibroblastic proliferation. The clinical features of FS vary depending on the phase in which the individual is present. The common clinical features are pain, and reduction in the range of motion in the capsular pattern. The available treatment options are medical therapy such as corticosteroid injection, physical therapy, joint mobilization, joint mobilization under anesthesia, and mirror therapy. When all the conservative methods fail then surgical procedures are used which include the surgical release of the restriction formed in the capsule. In conclusion, steroid injection along with physical therapy shows significant improvement in the range of motion and reduction in pain in the shoulder.

## Introduction and background

Frozen shoulder (FS) is a condition characterized by stiffness in the shoulder joint with or without a known cause [1]. Along with FS, frozen wrist, hip, and knee also exist. This stiff shoulder is caused either by a primary condition or is secondary to other conditions [1-5]. The etiology for the FS includes diabetes, thyroid involvement commonly hypothyroidism, nephrolithiasis, Parkinson's disease, cancer, shoulder injury, smoking, and neck surgeries. FS severely affects the individual’s life by hampering their activities of daily living and by leading to nocturnal discomfort [6].

The present literature shows the incidence of FS is two to five percent worldwide [7]. FS is commonly seen in the population lying in the age group 40-60. The incidence of FS is less common after the 70s except in certain cases with a traumatic shoulder injury [8]. The prevalence is more common in women than in men [4,9]. On the other hand, in the population suffering from the above-mentioned conditions such as diabetes, hypothyroidism, etc., the risk of FS is increased by 10% to 38% [8] Individuals suffering from type 1 diabetes are at the greatest risk whereas the risk further increases if the individual's age is more than 45 years. These patients are more prone to disability and greater reduction in the range of motion [7]. This is because of the increased level of glycated hemoglobin A1c (HbA1c) which is a deciding factor. Individuals with poor glycaemic control have a higher risk of developing FS [10].

Pathogenesis of FS

Clinical presentation of the FS includes pain, stiffness, and progressive loss of mobility of the shoulder joint. There is involvement of ligament and capsule and contracture that restricts the rotational interval of the affected shoulder. The nature of the FS can be described as proliferative, fibroblastic, and inflammatory [11]. In most cases, fibrotic changes are seen. Synovial biopsies indicate the presence of mast cells, macrophages, and T and B lymphocytes along with inflammatory mediators such as cytokines such as interleukin 1β, IL-6, IL-8, and tumor necrosis factor-alpha in the affected shoulder [12]. Presence of these inflammatory regulatory leads to dysregulation in collagen synthesis in the affected population.

Thickening of coracohumeral ligament is a prominent indication of FS, and its relation with supraspinatus and infraspinatus tendon contributes to the restriction of rotations of shoulder joint, Recently few studies showed minimal capsular restriction [13-15]. The purpose of the review is to increase knowledge about the current concepts and treatment strategies available.

## Review

FS is a biomechanical ailment that causes discomfort, limited mobility, and joint stiffness. Various types of conservative physiotherapy management are used in FS patients. The main aim of treatment is to reduce discomfort, recover, and regain range of motion. Heating modalities with exercises can be helpful in the treatment of FS patients. The mobilization exercises of the shoulder include pendulum exercises. Stretching can also be added to treatment. Strengthening exercises such as isometrics can be performed in later stages of FS.

Reduced use or non-use of certain body parts leads to anatomical reorganization in some parts of the brain, and neglect syndrome of the same part [16,17]. The less used part of the body can be easily damaged which produces a brain-based reaction leading to generation of muscle response which leads to FS [15]. A healthy glenohumeral joint offers great mobility and least stability but a reduced mobility leads to chronic hypoxia that already has low partial oxygen pressure which provides a suitable environment for the development of the inflammatory response which is mediated by factors like hypoxia-inducible factor-1 (HIF-1) and nuclear factor kappa B along with activation of several vascular and endothelial growth factors which are associated with inflammation, angiogenesis, and tissue destruction [18].

Oxidative stress associated with the presence of inflammatory cytokines in different parts of the shoulder ligament capsule complex may lead to increased accumulation of free radicals, which lead to subclinical alteration in both soft tissue and extracellular matrix [19,20].

Recent treatment modalities 

The treatment modalities are medical therapy, physical therapy, manipulations under anesthesia, nerve blockers, and steroid injections. Another option is surgical release of the capsular adhesions for improving range of motion and reducing pain. Surgical treatment is used when conservative treatment fails over a period of three to six months [21,22].

The management of FS changes with the progression of the disease; during the initial phase, which is the freezing phase, the duration is 13-36 weeks. Pain reduction and maintaining the available range of motion (ROM) is the mainstay of the treatment for pain; steroid injection and pendulum exercises are the choices of treatment [23].

The second stage is the frozen phase, 4-12 months. The main clinical features presented by the patients are reduction of the range of motion and moderate pain for which joint mobilization techniques such as Maitland, Mulligan, and Kaltenborn [24], can be used while in recent years newer mobilization technique is also found to be effective. A study conducted by Iqbal et al. in the year 2020 compared the effectiveness of the spencer technique and passive stretching in the patient with adhesive capsulitis; the individuals were in the age group 30-55 with idiopathic FS in stage 1 and stage 2 for at least 3 months. The result of the study suggests that the spencer technique is much more effective than passive stretching in individuals with FS [25].

For the individuals who are in the thawing stage of the FS, the duration of the thawing phase is 12-42 months; in this phase, progressive reduction of the pain and gradual increment in the range of motion are seen. A recent study showed that corticosteroid infiltration along with vigorous physical therapy provided the most benefit in the thawing phase of the FS [26].

A study was conducted by Louw et al. on 69 individuals who used mirror therapy. The patient was first asked to sit in front of the standing mirror and was asked to do movement of the unaffected shoulder, creating an illusion of movement in affected side. The movement was done 10 times. The individuals suffering from pain in the shoulder region with limited ROM found significant improvement in the ROM immediately after mirror therapy and a reduction in pain [27].

There are various manual therapy techniques that are proved to be effective in reducing pain as well as functional disability in subjects with FS. Depending upon the stage and intensity, the repetition and duration vary.

Maitland’s mobilization

Maitland mobilization reestablishes the gliding, rolling, and spinning motions of the two joints. Maitland performs accessory movement restoring the normal alignment of the joint. Physical functional activities are restored and joint stiffness and pain are reduced because of Maitland’s mobilization [24].

A study was performed on patients with FS in which the patients were treated with stretching. Another group underwent treatment of overhead pulley and virtual reality games for movements of the shoulder. After complete treatment, both groups were compared with each other. The results showed nearby equal effects of both studies [28]. Agarwal conducted a study that included patients with adhesive capsulitis; two different mobilization techniques were applied to the shoulder joint. Mobilization techniques included Kaltenborn mobilization and reverse distraction. Both techniques were helpful to reduce pain [29].

Almureef et al. showed the significance of manual therapy such as mobilization with traditional therapy for reducing the symptoms of adhesive capsulitis such as pain and improving the range of motion. After studying various randomized controlled trial studies, this review article was written by the author [30]. A study was carried out in individual patients of adhesive capsulitis with unilateral involvement of shoulder joint. For a three-month period, two different groups of patients were present in which, one patient population received low-intensity mobilizations and the other group received high-intensity mobilizations. In order to improve range of motion, high-intensity mobilization approaches are more effective according to the study [31].

Chen et al. conducted a study which showed the effectiveness of sensor instrument of movement in FS patients. This treatment should be advised in home programs and therapists will be able to give commands for treatment via telecommunications [32]. A Research on the effectiveness of movement with mobilization in adhesive capsulitis of the shoulder determined that mobilization can be an integral part of treatment for improving range of motion and reducing pain. The effect of mobilization is significant when it is included with a supervised exercise program [33]. Lee et al. conducted a study on patients of adhesive capsulitis; they use integrated Kinect with virtual reality and they introduced innovative treatment approach for FS rehabilitation. In this study, several shoulder movements were carried out in patients. After clinical examination, a significant effect of VR-based treatment on FS patients was found [34].

 Moon et al. conducted a study to check the effect of Maitland and Kaltenborn mobilization on subjects with FS. A total of 20 subjects were included in the study, Visual analog scale (VAS) and digital goniometer were used as outcome measures to assess pain and range of motion both pre- and post-intervention. The study concluded that there is no significant difference between Maitland and Kaltenborn’s mobilization but there was significant reduction in pain in both groups [35]. Chan et al. described various treatment approaches for FS. They described all the three stages of FS and the various treatment approaches of physical therapy in each stage. They concluded that physical therapy has significant effect on reducing pain and increasing the range of motion in patients with FS [36].

Hammad et al. conducted a study in which they compared the effect of Kaltenborn and Kaltenborn combined with thermotherapy. They used shoulder pain and disability index (SPADI) as an outcome measure to measure the pain and disability pre- and post-treatment. They concluded that Kaltenborn combined with Thermotherapy is more effective as compared to Kaltenborn alone [37]. Duzgun et al. checked the immediate effect of posterior capsule stretching and scapular mobilization on subjects with FS. They concluded that posterior capsule stretching has a positive effect on the shoulder range of motion [38].

Tedla et al. conducted a systematic review and meta-analysis on the effect of proprioceptive neuromuscular inhibition (PNF) in subjects with FS. They studied 410 studies of which 10 articles were included in the study. They concluded that PNF is superior to conventional physiotherapy in order to decrease pain and increase the range of motion [39]. Thu et al. compared the effect of ultrasound-guided platelet-rich plasma (PRP) and conventional physiotherapy. They included 64 subjects with FS. They used visual analog scale and passive ROM as outcome measures. They concluded that PRP is a useful option in treating patients with FS [40].

Shabbir et al. checked the effectiveness of PNF and conventional physiotherapy in treatment of FS. They concluded that PNF along with conventional physiotherapy is a better treatment option for FS [41]. Razzaq et al. compared Mulligan’s mobilization and muscle energy technique for subjects with FS. They included 70 subjects and used numeric pain rating scale (NPRS), SPADI, and ROM as outcome measures. They concluded that both have an effect in reducing pain and increasing ROM [42].

Mohamed et al. conducted a randomized control trial to see the effect of dynamic scapular recognition exercises on subjects with FS. The treatment was given for two weeks and SPADI and ROM were the outcome measures used. They concluded that there is a significant difference between dynamic scapular recognition exercises and conventional physiotherapy [43]. A summary of the articles reviewed is provided in Table 1.

**Table 1:** Summary of articles reviewed VAS = visual analogue scale; SPADI = shoulder pain and disability index; ROM = range of motion; PNF = proprioceptive neuromuscular facilitation; PRP = platelet-rich plasma; DASH = disabilities of the arm, shoulder and hand; MET = muscle energy technique; MWM = mobilization with movement; NPRS = numerical pain rating scale; FS = frozen shoulder

Sr no	Year	Author	Intervention	Outcome	Intervention period	Result	Analysis
1	2015	Moon et al. [35]	Maitland mobilization and Kaltenborn mobilization	VAS and digital goniometer	three times per week for four weeks	There were differences in pain (p < 0.05).	Maitland mainly focuses on the joint whereas Kaltenborn concentrates more on the capsule.
2	2017	Chan et al. [36]	Stretching, Codman exercises, strengthening	Pain, stiffness	26 months	Physical therapy reduced pain and stiffness in the shoulder joint.	Physiotherapy includes pain relief and strengthening approaches, both helpful in subjects with FS.
3	2019	Hammad et al. [37]	Kaltenborn and thermotherapy	SPADI	three times/week on alternative days for 3 weeks	A combination of thermotherapy and Kaltenborn was found to be more effective.	Thermotherapy increases the local blood circulation and helps in the healing process whereas Kaltenborn helps in restoring the range of motion.
4	2019	Duzgun et al. [38]	scapular mobilization, manual posterior capsule stretching	ROM	immediate effect	Manual posterior capsule interventions and scapular mobilization were beneficial in FS patients.	Posterior capsule tightness is a sign of FS mobilization and capsule intervention is helpful.
5	2019	Tedla et al. [39]	PNF	Pain, ROM, and disability		PNF is superior to conventional physical therapy.	PNF provides proprioceptive facilitation to the shoulder joint. Proprioception is the most missed component in the shoulder. So, PNF helps restore range.
6	2020	Thu et al. [40]	Ultrasound Guided PRP and conventional physiotherapy	VAS, shoulder passive ROM	three times/week for six weeks	Platelet-rich plasma is a beneficial option for treating patients with AC.	Platelet-rich plasma is a great treatment option as it promotes healing and hence reduces pain.
7	2021	Shabbir et al. [41]	PNF and conventional therapy	DASH questionnaire, SPADI, and goniometer	one month	Proprioceptive exercises are more effective as compared to conventional physical therapy alone.	
8	2022	Razzaq et al. [42]	MET and MWM	Numeric pain rating scale, goniometer and shoulder pain, and disability index.	two sets per session three days a week for three weeks	MET and MWM were both found to be effective.	The muscle energy technique uses both strengthening and stretching in a combination whereas Mulligan works on micro malalignment.
9	2020	Iqbal et al. [25]	Spencer technique and passive stretching.	NPRS, goniometer, SPADI	three sessions per week on alternate days for four weeks	Spencer's technique was found to be more effective than passive stretching in treating patients with adhesive capsulitis.	Spencer’s technique helps in reducing restriction in movement.
10	2020	Mohamed et al. [43]	Dynamic scapular recognition exercises	SPADI, range of motion	two weeks	Significant effects of dynamic scapular recognition exercises were seen.	Dynamic scapular exercises concentrate on the scapula as scapular muscle weakness is seen in FS.

## Conclusions

We conclude that FS requires a multidisciplinary approach to gain early recovery; the treatment is selected based on the phase in which the individuals report to the medical practitioner. The mainstay of the treatment includes steroid injection along with joint mobilization exercises; the mobilization techniques used for improving range of motion and reducing restriction and decreasing pain are Mulligan, Kaltenborn, Maitland, and Spencer techniques. When the conservative treatment fails, one can opt for surgical treatment which consists of mobilization of the shoulder joint under anesthesia or more severe cases require surgical release of the capsular restriction. It significantly improves ROM and reduces pain. Another conservative treatment used for the treatment of the FS is mirror therapy which helped individuals to improve their ROM and reduce pain by making them stand in front of the mirror and asking them to perform the movement. 
